# Endoscopic ultrasound-guided gallbladder drainage for distal malignant biliary obstruction: Outcomes from a multicenter cohort

**DOI:** 10.1055/a-2631-7857

**Published:** 2025-07-23

**Authors:** Michael Chieng, Tara Fox, Jerry Yung-Lun Chin, Estella Johns, Rees Cameron, Frank Weilert

**Affiliations:** 1 3714 Gastroenterology, Waikato District Health Board, Hamilton, New Zealand; 2 8493 Gastroenterology, Wellington Regional Hospital, Newtown, New Zealand

**Keywords:** Endoscopic ultrasonography, Biliary tract, Intervention EUS, Pancreatobiliary (ERCP/PTCD), Strictures

## Abstract

**Background and study aims:**

Endoscopic ultrasound-guided gallbladder drainage (EUS-GBD) is described as salvage therapy for patients with distal malignant biliary obstruction (DMBO). However, there is a paucity of data reporting on clinical outcomes for this indication.

**Patients and methods:**

A multicenter retrospective review of 26 EUS-GBD cases was performed between 2017 and 2023 at two centers in New Zealand. Efficacy outcomes of technical success (TS), clinical success (CS), length of stay (LOS), and resumption of cancer therapy were recorded. Adverse events (AEs), reinterventions, time to reintervention, and survival also were examined. Reinterventions were categorized into early (< 7 days) or delayed procedures (≥ 7 days).

**Results:**

Mean age was 74 years. Pancreatic cancer was the most common diagnosis. All included cases were unresectable and eight of 26 (30.8%) were chemotherapy candidates. TS and CS were achieved in all patients. At 14 days, bilirubin decreased from a mean of 139 to 55 μmol/L, a 60.4% reduction from baseline value. Mean LOS was 3 days. Of eligible patients, 87.5% were able to resume chemotherapy post-procedure. There were no intra-procedural complications nor early reinterventions. Four serious AEs (15.4%) required reintervention; the remaining nine were treated conservatively. Median survival was 103 days.

**Conclusions:**

EUS-GBD is a clinically effective salvage therapy for DMBO that may be positioned after unsuccessful endoscopic retrograde cholangiopancreatography or EUS-BD in a single anesthetic session. Most patients have a short LOS and few serious AEs. Furthermore, oncologic therapy can be successfully resumed post-procedure. EUS-GBD, therefore, should be considered an effective, safe, and durable addition to the treatment armamentarium for DMBO.

## Introduction

Distal malignant biliary obstruction (DMBO) is a serious diagnosis that can result in significant morbidity and mortality in patients due to development of jaundice and related complications. Addressing this condition is critical to improving quality of life and survival outcomes, especially in non-surgical candidates with advanced oncologic disease. Established drainage methods include endoscopic retrograde cholangiopancreatography (ERCP) and endoscopic ultrasound-guided biliary drainage (EUS-BD) 1 2 . However, biliary access to either the papilla or the bile duct can be challenging due to distorted anatomy from underlying malignancy. Endoscopic ultrasound-guided gallbladder drainage (EUS-GBD), therefore, has emerged as an innovative and potentially effective salvage therapy for patients with DMBO in whocm these conventional approaches fail 3 4 .

The 2024 American Society for Gastrointestinal Endoscopy (ASGE) guidelines suggest EUS-BD techniques can be grouped into EUS-guided choledochoduodenostomy (EUS-CDD), hepaticogastrostomy (EUS-HGS), and/or rendezvous techniques 5 . These approaches each have individual challenges and may not be feasible for a proportion of patients. For example, EUS-CDD with a lumen-apposing metal stent (LAMS) requires a dilated common bile duct (CBD) for safe deployment of the distal flange, given the perpendicular approach path 6 7 . EUS-CDD also requires an unobstructed sonographic window, free from interposing vessels 8 . EUS-HGS, on the other hand, is challenging from a technical perspective, and there is a lack of dedicated equipment designed for this technique 9 . Consequences of maldeployment are accompanied by risks due to relative proximity to the mediastinum. Furthermore, bile leak and stent migration, particularly from the proximal end of the stomach, have been described 10 . Rendezvous procedures are contingent on successful guidewire access across the ampulla, which may not be possible due to tumor invasion or anatomical distortion 11 . When these biliary access techniques are exhausted, percutaneous biliary drainage (PT-BD) is typically utilized as the final salvage option. However, PT-BD is also associated with several well-documented shortcomings including tube displacement, infections, bile leakage, and patient discomfort 12 . Considering this combination of factors in aggregate, there is a clear clinical need for alternatives in a subset of patients with DMBO.

EUS-GBD leverages the proximity of the gallbladder to the gastrointestinal lumen to establish a new passage for biliary drainage. This offers a viable and potentially safer alternative to the aforementioned EUS-BD techniques, particularly when the gallbladder is distended 13 . Recent studies have shown technical success rates for EUS-GBD exceeding 95%, and clinical success rates of more than 85%, coupled with lower adverse events (AEs) compared with other advanced options 4 14 . EUS-GBD also can be positioned as a sequential therapeutic option immediately after unsuccessful attempts at ERCP or EUS-BD, meaning a single procedure session can guarantee biliary drainage for any individual patient 5 . This reduces cumulative procedure and anesthetic exposures for patients who are defined by high-surgical-risks and poor anesthetic candidacy due to their underlying malignancy.

Despite this promise, evidence for EUS-GBD in DMBO largely derives from small, retrospective series or single-center studies, which limits the applicability of findings to broader patient populations. Our multicenter analysis evaluated the efficacy, safety, and longer-term outcomes of EUS-GBD performed for DMBO in non-surgical patients across two tertiary referral hospitals in New Zealand. We also report data related to resumption of cancer therapy, which has direct clinical relevance aligned with the broader patient trajectory. By addressing this gap in the literature, our study aimed to provide additional evidence to guide clinical decision-making in this cohort.

## Patients and methods

### Study design and outcomes

We performed a multicenter, retrospective cohort study at two tertiary referral hospitals in New Zealand. The study aimed to evaluate outcomes of EUS-GBD performed for DMBO indications. Efficacy outcomes were technical success rates, clinical success rates, length of hospital stay, and proportion of patients able to resume oncologic treatments. Safety outcomes included AEs, reinterventions, and patient survival. Follow-up duration was from time of intervention until patient death.

### Outcome definitions

Technical success was defined as successful deployment of a stent with directly visualized bile drainage during the procedure. Clinical success was defined as improvement in bilirubin and/or jaundice within 72 hours of the procedure. Bilirubin was also recorded at 14 days as a durable efficacy measure. Eligibility for post-procedure oncologic therapy was performed within independent oncologic clinics considering oncologic factors, comorbidity, and functional capacity

AEs and reinterventions were categorized as early (< 7 days) or delayed (≥ 7 days) and according to the ASGE lexicon. These included bile leak, stent migration, bleeding, perforation, infection and death.

### Patient selection

Included participants were adults (≥ 18 years) diagnosed with DMBO who underwent EUS-GBD as part of their clinical management between January 2017 and December 2023.

Eligibility criteria included: 1) unsuccessful endoscopic retrograde cholangiopancreatography (ERCP) and/or EUS-BD due to anatomical or technical challenges; 2) unsuitability for percutaneous transhepatic cholangiography due to patient preference or technical considerations; and 3) presence of a distended and accessible gallbladder confirmed by imaging.

Exclusion criteria included patients with cystic duct obstruction or inability to tolerate anesthesia. Cystic duct obstruction was assessed via pre-procedural cross-sectional imaging and at time of diagnostic EUS prior to GBD. Patients with incomplete data regarding cancer diagnosis, treatment, and/or survival were excluded from analyses.

At both centers, patients were prospectively consented for the possibility of ERCP, EUS-BD, and/or EUS-GBD prior to administration of an aesthetic so that any procedure could be performed in a single anesthetic session. Local ethics approval was provided at both participating centers before commencement.

### Procedure details

All EUS-GBD procedures were performed by experienced interventional endoscopists using an aesthetic-assisted sedation including propofol or general anesthetic. The procedure utilized linear echoendoscopes to identify the gallbladder and a vessel-free path, followed by electrocautery enhanced LAMS puncture through the duodenal wall using a freehand technique. The distal end of the LAMS was deployed within the gallbladder and the proximal end within the duodenum. All of the LAMS were the AXIOS stent manufactured by Boston Scientific. Size was determined at the discretion of the individual endoscopists. Placement of additional co-axial plastic pigtail stents was also performed according to proceduralist preferences.

### Data collection

Patient demographics, baseline clinical characteristics, and procedure details were collected from electronic medical records. Only data from patients who underwent EUS-GBD procedures were collected.

### Statistical analysis

For continuous variables with normal data distribution, means (standard deviations) are reported; for non-normally distributed data medians (interquartile ranges [IQRs]) are used. Categorical variables are presented using frequencies (percentages).

## Results

### Participant characteristics

Twenty-six patients with DMBO were included in the study. Participant characteristics are presented in Table 1 . Mean age was 74 years (SD = 10 years) with nine (34.6%) females and 17 males (65.4%). Pancreatic malignancy was the most common diagnosis, accounting for 76.9% of cases (20/26). All cancers were advanced (either locally or metastatic) and unresectable. Of the patients, 30.8% (8/26) were eligible candidates for chemotherapy. Average Charlson Comorbidity Index was 10 and mean American Society of Anesthesiologists (ASA) score was 4. The reason for failure of prior ERCP and/or EUS-BD included inaccessible papilla (13/26), inability to cannulate the CBD (9/26), maldeployment of prior CDD (2/26), and not otherwise specified (2/26).

**Table:** **Table 1** Patient characteristics, oncologic details, and comorbidity.

Parameter	EUS-GBD, n (%)
Age, years	74 ± 10
Gender	
Female	9 (34.6)
Male	17 (65.4)
Ethnicity	
Māori (indigenous)	5 (19.2)
European	16 (61.5)
Other	5 (19.2)
Malignancy type	
Pancreatic cancer	20 (76.9)
Cholangiocarcinoma	2 (7.7)
Ampullary cancer	1 (3.8)
Other	3 (11.5)
Comorbidity	
Charlson Comorbidity Index	9.5 ± 1.8
ASA physical status classification	3.8 ± 0.8
Data are presented as mean ± standard deviation or number of participants (% of participants). ASA, American Society of Anesthesiologists; EUS-GBD, endoscopic ultrasound-guided biliary drainage.	

### Efficacy outcomes

Efficacy outcomes are presented in Table 2 . Technical and clinical success was achieved in all 26 cases (100%). The LAMS size ranged from 8 mm to 15 mm, with the 10-mm stent being the most commonly used (20/26, 76.9%). Co-axial plastic pigtail stents were placed in 26.9% (7/26). Mean pre-procedure bilirubin was 139 µmol/L (SD = 117 µmol/L) and improved to 70 µmol/L (SD = 50 µmol/L) at 72 hours. After 14 days, the mean bilirubin was 55 µmol/L (SD = 40 µmol/L), equivalent to an average reduction in bilirubin of 84 µmol/L or 60.4% from baseline value. The bilirubin fully normalized in five patients (19.2%) over the follow-up period. Mean length of hospital stay after intervention was 3 days (SD = 3). Of the eight patients who were deemed to be chemotherapy candidates, only one (12.5%) was ineligible to receive systemic treatment post-procedure.

**Table:** **Table 2** Efficacy outcomes.

Parameter	EUS-GBD n (%)
Technical success	26 (100)
Clinical success	26 (100)
LAMS size	
8 mm	5 (19.2)
10 mm	20 (76.9)
15 mm	1 (3.8)
Co-axial plastic stents	
Yes	7 (26.9)
No	19 (73.1)
Biochemistry	
Pre-procedure bilirubin	139 ± 117 µmol/L
Post-procedure bilirubin at 72hrs	70 ± 50 µmol/L
Change in bilirubin at 72 hrs (△72H)	-63 µmol/L
Post-procedure bilirubin at 14 days	55 ± 40 µmol/L
Change in bilirubin at 14 days (△14D)	-84 µmol/L
Length of stay	
Days	3 ± 3
Eligible to resume cancer therapy	n = 8
Yes	7 (87.5)
No	1 (12.5)
Data are presented as mean ± standard deviation or number of participants (% of participants). EUS-GBD, endoscopic ultrasound-guided biliary drainage; LAM, lumen-apposing metal stent	

### Safety outcomes

Safety outcome data relating to AEs, reinterventions, and survival are presented in Table 3 . There were no intra-procedural AEs, early AEs, nor early reinterventions recorded. Delayed AEs (> 7 days) were observed in 13 patients (50%), of which four were classified as serious. There was one case of cholangitis (1/26, 3.85%) and three cases of recurrent biliary obstruction (3/26, 11.5%). There were no bile leak, stent migration, bleeding, or peritonitis AEs recorded. All the non-serious AEs were infections and were suspected to be hepatobiliary in origin due to the microorganisms cultured (gram negative and/or anerobic bacteria) and biochemical results (deranged liver biochemistry with otherwise unremarkable septic screening). Each resolved with antibiotic therapy alone and did not require any invasive intervention (9/26, 34.6%).

**Table:** **Table 3** Safety outcomes: Adverse events, reinterventions, and survival.

Parameter	EUS-GBD n (%)
Procedure complications	
Yes	0 (0)
No	26 (100)
Early adverse events (≤ 7 days)	
Yes	0 (0)
No	26 (100)
Early reinterventions	
Yes	0 (0)
No	26 (100)
Delayed adverse events (> 7 days)	
Yes	13 (50)
Serious adverse event	4 (15.4)
Cholangitis	1 (3.8)
Recurrent biliary obstruction	3 (11.5)
Non-serious AE	9 (34.6)
Hepatobiliary infections	9 (34.6)
No	13 (50)
Delayed Reinterventions	
Yes	4 (15.4)
ERCP	1 (3.9)
Percutaneous drainage (PT-BD)	3 (11.5)
No	22 (84.6)
Survival	
Days	103 (38–192)
Data are presented as mean ± standard deviation, median (interquartile range), or number of participants (% of participants). ERCP, endoscopic retrograde cholangiopancreatography; EUS-GBD, endoscopic ultrasound-guided biliary drainage.	

Four patients (15.4%) with serious AEs (SAEs) required reintervention. The case that was complicated by cholangitis had a repeat attempt at ERCP which was successful and the three cases of recurrent biliary obstruction were managed with percutaneous biliary drainage (PT-BD). These procedures occurred at 14 days for the ERCP; 20 days, 30 days and 121 days for the PT-BD reinterventions. Median time to reintervention was 25 days. Each of these cases only required one reintervention procedure. Median survival of the cohort was 103 days (IQR 38–192).

## Discussion

Our study highlights excellent technical and clinical efficacy of utilizing EUS-GBD as a salvage therapy for DMBO after unsuccessful attempts at conventional techniques. Although this technique is being successfully deployed for non-surgical candidates with cholecystitis 15 16 , the findings in our study add weight to the growing body of evidence suggesting efficacy and safety for DMBO indications as well. We have demonstrated an acceptable profile of reintervention data for a pre-selected population of highly comorbid patients, and durability of the intervention over these patients’ survival duration.

Our findings show an approximate halving of serum bilirubin level within 3 days and a 60% reduction within 2 weeks. This represents a rapid and significant reduction in jaundice, underscoring the effective drainage established via the cystic duct to LAMS channel in obstructed biliary systems. This is highlighted by the fact that almost one-fifth of patients achieved full normalization of their serum bilirubin during the follow-up period. The tempo of this change is relevant for patients with biliary-associated malignancy, where timely resolution of obstruction can reduce jaundice-associated symptoms, improve liver function, and enable resumption of systemic oncologic therapy. In our cohort, the vast majority of patients who were candidates for chemotherapy were eligible to resume this post-procedure. Other studies have also demonstrated significant early reductions in serum bilirubin following successful EUS-GBD 17 18 . Although mean bilirubin levels improved significantly in our cohort, it is notable that full normalization was not achieved in all patients, likely due to residual tumor burden and associated hepatic dysfunction. For patients with limited anticipated survival duration, this underscores the importance of setting realistic prospective goals.

Our efficacy findings are similar to those reported in the literature, including systematic reviews (SRs) by McDonagh and Osman et al. 19 20 . The review by McDonagh et al. included seven studies with 136 patients, and Osman et al. included 15 studies with 161 patients undergoing EUS-GBD for malignant biliary obstruction indications. The authors reported pooled technical success rates of 100% and 92.1%, respectively, with slightly lower clinical success rates of 85% and 81.62%, respectively. Of note, our relatively higher clinical success rates would remain unchanged applying the same definitional criteria as both reviews (decrease in serum bilirubin > 50%, normalization of serum bilirubin, and/or improvement in jaundice/symptoms within 2 weeks post-procedure). Of interest, the recent multicenter Spanish study by Martinez-Moreno et al. reported greater proportions of bilirubin normalization (65.6%) compared with our cohort (17.9%). However, they also had more patients eligible for surgery (17.7% vs 0%) and oncologic treatment (80.2% vs 30.8%), which likely reflects differences in the included populations and underlying cancer status 18 . Of those eligible for chemotherapy in our cohort, a greater proportion were able to resume this post-procedure (87.5% vs 57.1%), perhaps also reflecting differences in candidacy selection for these systemic treatments in the adjuvant setting 18 .

A key advantage of expanding the range of therapeutic EUS techniques for DMBO lies in the ability to complement ERCP to ensure successful biliary drainage for patients in a single anesthetic session. In our study, EUS-GBD served as a rescue therapy in a challenging case in which stent maldeployment occurred during a previously attempted EUS-CDD. This highlights the versatility of EUS-GBD as a technically straightforward salvage option, compared with EUS-HGS, for example. Our findings also support the notion that sequential ERCP and EUS-GBD represents a streamlined approach. Much has been discussed previously about the importance of demonstrable cystic duct patency for EUS-GBD to be therapeutically viable 21 . In a historic retrospective study assessing incidence of cystic duct patency on cholangiograms in patients with MBO, only 50% of participants had a patent hepato-cystic junction 22 . Our study cohort was pre-selected based on cystic duct patency and, therefore, the total denominator including patients ineligible for this reason is not known in our population. This should be a key metric to capture in future prospective studies because it may be one of the limitations to broader adoption in other settings.

When comparing large series of first-line ERCP versus first-line EUS-BD (including EUS-GBD) for DMBO, there appear to be comparable outcomes (technical success: ERCP 92.66% vs EUS-BD 92.79%, clinical success: ERCP 93.2% vs EUS-BD 93.4%) 20 23 . Subgroup analyses of specific EUS-GBD AEs and reinterventions show superiority to ERCP by 10% to 15% and parity to other EUS-BD AEs, with only a 1% to 2% difference in these outcomes. The GALLBLADEUS Study compared EUS-CDD with EUS-GBD in the second-line setting for DMBO after failed ERCP, and found that EUS-GBD was non-inferior with regards to technical success and clinical success 4 . This was coupled with a statistically significantly lower rate of late morbidity for EUS-GBD (7.3% vs 21.6%, *P* = 0.042) and no difference in survival or recurrent obstruction 4 . This creates a strong narrative for adoption of EUS-GBD into clinical practice as either a second- or third-line therapy 6 .

Our total AE rate of 50% was higher than the aforementioned SRs but serious AEs were similar (~13% in SRs vs 15.4% in our cohort) 19 20 . McDonagh et al. did not report non-stent-related infectious complications, and only included AEs related to peritonitis, bleeding, bile leakage, stent migration, and stent occlusion. Osman et al. also reported only one case of cholangitis in 10 studies. In contrast, most of our AEs were infectious in nature and presumed to be biliary in origin. These were captured from thorough review of electronic health records from time of intervention until patient death. Almost all infections, excluding one case of cholangitis, were treated conservatively with antibiotics. For the small proportion of patients who did require reintervention, only one additional procedure was necessary in all cases.

With regard to co-axial plastic stents, 26.9% had these placed at the time of EUS-GBD. Co-axial stenting is theorized to improve LAMS patency, stabilize LAMS position, prevent tissue ingrowth, and protect against LAMS abrasion with the gallbladder mucosa 16 . In a multicenter US study of EUS-CDD, there were significantly higher reinterventions performed for patients that underwent LAMS alone versus LAMS plus additional co-axial stenting (50% vs 5%, *P* = 0.02) 24 . These findings were not consistent with a subsequent multicenter study by Garcia-Sumalla et al., however, who found that co-axial plastic stents made no difference with regard to clinical success, AEs, nor recurrent biliary obstruction. Plastic stent placement also significantly increased procedure time 25 . A prospective, multicenter, randomized controlled trial has begun and should hopefully provide a more definitive conclusion to this question 26 .

There are inherent limitations to our study, which include the retrospective design and unknowns regarding the total denominator of patients with unsuccessful ERCP/EUS-BD over the study period. Both of these factors may impact generalizability of results. Furthermore, variations in endoscopist experience and procedure techniques across centers may introduce bias.

## Conclusions

Overall, our study highlights the efficacy of EUS-GBD with regard to meaningful improvements in jaundice and ability to resume oncologic therapies in DMBO settings. This technique can be successfully positioned after attempts at ERCP or EUS-BD within a single anesthetic session. Our findings demonstrate rapid and significant reduction in bilirubin, low rates of AEs, reinterventions, and peri-procedural morbidity and mortality.
